# *catena*-Poly[[tribenzyl­tin(IV)]-μ-2-(piperidin-1-ylcarbothio­ylsulfan­yl)acetato-κ^2^ *O*:*O*′]

**DOI:** 10.1107/S1600536810035221

**Published:** 2010-09-08

**Authors:** Thy Chun Keng, Kong Mun Lo, Seik Weng Ng

**Affiliations:** a Department of Chemistry, University of Malaya, 50603 Kuala Lumpur, Malaysia

## Abstract

The Sn atom in the title polymeric compound, [Sn(C_7_H_7_)_3_(C_8_H_12_NO_2_S_2_)]_*n*_, exists in a *trans*-C_3_O_2_ trigonal-bipyramidal coordination environment in the two independent formula units. The carboxyl­ate moiety functions in a bridging mode, linking adjacent triorganotin cations into a linear chain running along the shortest axis of the triclinic unit cell; the repeat distance of the polymer is half the *a*-axial length. In one formula unit, the Sn atom is displaced out of the equatorial plane in the direction of the Sn—O_covalent_ bond by 0.093 (4) Å and in the second mol­ecule, the Sn atom is displaced by 0.105 (4) Å in the same direction.

## Related literature

Trialkyl­tin carboxyl­ates are generally carboxyl­ate-bridged polymers, see: Ng *et al.* (1988 ▶). For the direct synthesis of substituted tribenzyl­tin chlorides, see: Sisido *et al.* (1961 ▶). For the synthesis of dithio­carbamoyl­acetic acids, see: Nachmias (1952 ▶). For background to the triorganotin derivatives of dithio­carbamyl­acetic acids, see: Ng & Kumar Das (1991 ▶).
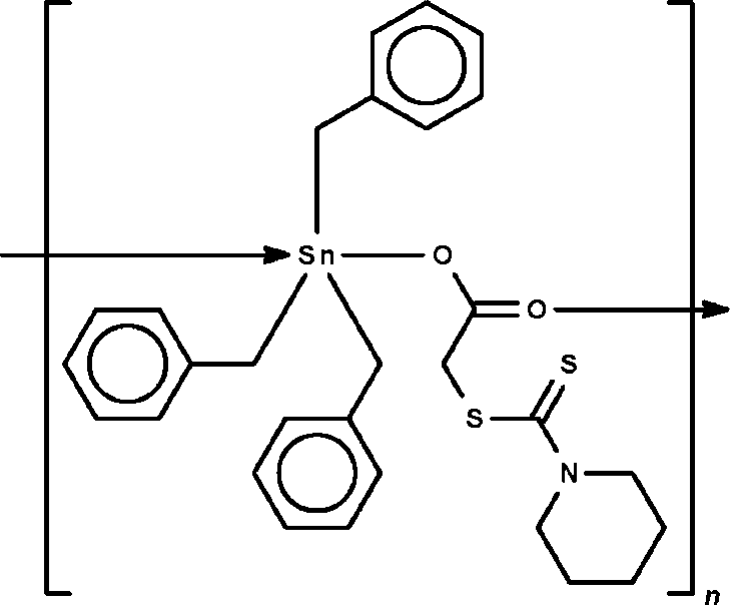


## Experimental

### 

#### Crystal data

[Sn(C_7_H_7_)_3_(C_8_H_12_NO_2_S_2_)]
*M* *_r_* = 610.37
Triclinic, 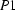

*a* = 10.7500 (7) Å
*b* = 11.3594 (7) Å
*c* = 12.5494 (8) Å
α = 79.494 (1)°
β = 81.890 (1)°
γ = 75.294 (1)°
*V* = 1450.15 (16) Å^3^
*Z* = 2
Mo *K*α radiation
μ = 1.05 mm^−1^
*T* = 293 K
0.30 × 0.20 × 0.10 mm

#### Data collection

Bruker SMART APEX diffractometer
Absorption correction: multi-scan (*SADABS*; Sheldrick, 1996 ▶) *T*_min_ = 0.744, *T*_max_ = 0.902
13931 measured reflections
11121 independent reflections
9448 reflections with *I* > 2σ(*I*)
*R*_int_ = 0.020

#### Refinement

*R*[*F* ^2^ > 2σ(*F* ^2^)] = 0.035
*wR*(*F* ^2^) = 0.095
*S* = 1.03
11121 reflections
559 parameters
89 restraints
H-atom parameters constrained
Δρ_max_ = 0.75 e Å^−3^
Δρ_min_ = −0.41 e Å^−3^
Absolute structure: Flack (1983 ▶), 4513 Friedel pairs
Flack parameter: −0.01 (2)

### 

Data collection: *APEX2* (Bruker, 2009 ▶); cell refinement: *SAINT* (Bruker, 2009 ▶); data reduction: *SAINT*; program(s) used to solve structure: *SHELXS97* (Sheldrick, 2008 ▶); program(s) used to refine structure: *SHELXL97* (Sheldrick, 2008 ▶); molecular graphics: *X-SEED* (Barbour, 2001 ▶); software used to prepare material for publication: *publCIF* (Westrip, 2010 ▶).

## Supplementary Material

Crystal structure: contains datablocks global, I. DOI: 10.1107/S1600536810035221/bt5336sup1.cif

Structure factors: contains datablocks I. DOI: 10.1107/S1600536810035221/bt5336Isup2.hkl

Additional supplementary materials:  crystallographic information; 3D view; checkCIF report

## Figures and Tables

**Table:** 

Sn1—C1	2.146 (5)
Sn1—C8	2.153 (5)
Sn1—C15	2.152 (5)
Sn1—O1	2.268 (3)
Sn1—O4^i^	2.276 (3)
Sn2—C22	2.144 (5)
Sn2—C29	2.146 (5)
Sn2—C36	2.143 (5)
Sn2—O2	2.272 (3)
Sn2—O3	2.277 (4)

**Table:** 

O1—Sn1—O4^i^	174.2 (1)
O2—Sn2—O3	174.4 (1)

Symmetry code: (i) 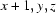.

**Fig. 1.**
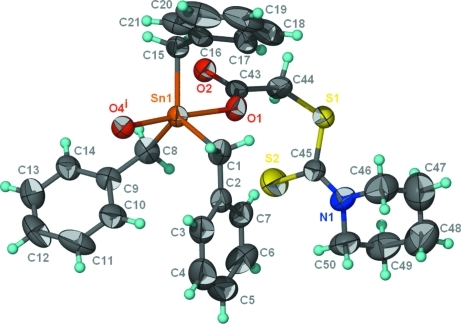
Anisotropic displacement ellipsoid plot (Barbour, 2001) of one of the two independent formula of Sn(C7H7)3(C8H12NO2S2) at the 50% probability level. Hydrogen atoms are drawn as spheres of arbitrary radius.

**Fig. 2.**
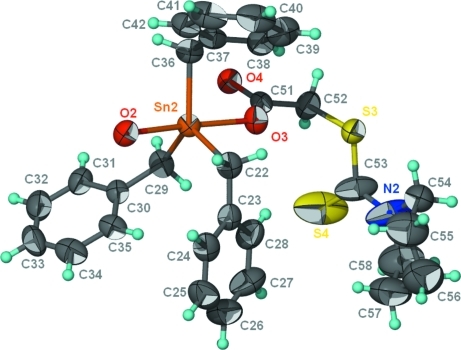
Anisotropic displacement ellipsoid plot (Barbour, 2001) of the second formula unit of Sn(C7H7)3(C8H12NO2S2) at the 50% probability level. Hydrogen atoms are drawn as spheres of arbitrary radius. The symmetry code is given in Table 1.

**Fig. 3.**
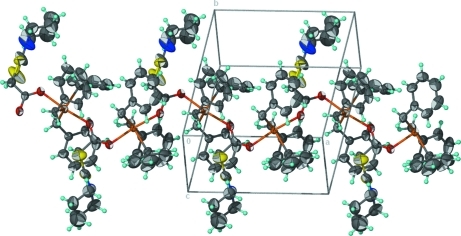
Anisotropic displacement ellipsoid plot (Barbour, 2001) of the chain structure of polymeric Sn(C7H7)3(C8H12NO2S2) at the 50% probability level. Hydrogen atoms are drawn as spheres of arbitrary radius.

## supplementary crystallographic information

### Comment

Trialkyltin carboxylates are generally exists as carboxylate-bridged polymers (Ng *et al.*, 1988). We have reported the triorganotin derivatives of dithiocarbamylacetic acids, a class plant growth hormones (Ng & Kumar Das, 1991). The title tribenzyltin derivative crystallizes as two independent formula units; both have their tin atoms in a *trans*-C_3_SnO_2_ trigonal bipyramidal coordination environment (Figs. 1 and 2.) The carboxylate portion functions in a bridging mode to link adjacent triorganotin cations into a linear chain running along the shortest axis of the triclinic unit cell (Fig. 3).

### Experimental

Pentamethylenedithiocarbomylacetic acid was synthesized from piperidine, carbon disulfide and chloroacetic acid (Nachmias, 1952). Tribenzyltin chloride was prepared by direct synthesis from benzyl chloride and tin powder in a mixture of toluene and water (Sisido *et al.*, 1961). The triorganotin chloride was hydrolyzed with dilute sodium hydroxide solution to give tribenzyltin hydroxide. The carboxylic acid (0.14 g, 0.73 mmol) and the organotin hydroxide (0.30 g, 0.73 mmol) were heated in ethanol (100 ml) for 2 hours. After filtering the mixture, colorless crystals were obtained upon slow evaporation of the filtrate.

### Refinement

Hydrogen atoms were placed at calculated positions (C–H 0.93–0.97 Å) and were treated as riding on their parent atoms, with *U*(H) set to 1.2 times *U*_eq_(C).

The aromatic rings of the benzyl chains were refined as rigid hexagons of 1.39 Å sides. The carbon–carbon bond distances in the piperidinyl rings were tightly restrained to 1.500+0.005 Å and the 1,3-related carbon–carbon distances to 2.350+0.005 Å. The anisotropic displacement parameters of the carbon atoms of the piperidinyl rings were restrained to be nearly isotropic.

In the dithiocarbamato C_2_N–C fragment, the nitrogen–carbon_sulfur_ distances were restrained to 1.35±0.01 Å and the nitrogen–carbon_methylene_ distances to 1.45±0.01 Å.

### Crystal data

**Table:**

[Sn(C_7_H_7_)_3_(C_8_H_12_NO_2_S_2_)]	*Z* = 2
*M**_r_* = 610.37	*F*(000) = 624
Triclinic, *P*1	*D*_x_ = 1.398 Mg m^−^^3^
Hall symbol: P 1	Mo *K*α radiation, λ = 0.71073 Å
*a* = 10.7500 (7) Å	Cell parameters from 6011 reflections
*b* = 11.3594 (7) Å	θ = 2.3–24.8°
*c* = 12.5494 (8) Å	µ = 1.05 mm^−^^1^
α = 79.494 (1)°	*T* = 293 K
β = 81.890 (1)°	Wedge, yellow
γ = 75.294 (1)°	0.30 × 0.20 × 0.10 mm
*V* = 1450.15 (16) Å^3^	

### Data collection

**Table:**

Bruker SMART APEX diffractometer	11121 independent reflections
Radiation source: fine-focus sealed tube	9448 reflections with *I* > 2σ(*I*)
graphite	*R*_int_ = 0.020
ω scans	θ_max_ = 27.5°, θ_min_ = 1.9°
Absorption correction: multi-scan (*SADABS*; Sheldrick, 1996)	*h* = −13→13
*T*_min_ = 0.744, *T*_max_ = 0.902	*k* = −14→13
13931 measured reflections	*l* = −16→15

### Refinement

**Table:**

Refinement on *F*^2^	Secondary atom site location: difference Fourier map
Least-squares matrix: full	Hydrogen site location: inferred from neighbouring sites
*R*[*F*^2^ > 2σ(*F*^2^)] = 0.035	H-atom parameters constrained
*wR*(*F*^2^) = 0.095	*w* = 1/[σ^2^(*F*_o_^2^) + (0.0488*P*)^2^ + ] where *P* = (*F*_o_^2^ + 2*F*_c_^2^)/3
*S* = 1.03	(Δ/σ)_max_ = 0.001
11121 reflections	Δρ_max_ = 0.75 e Å^−^^3^
559 parameters	Δρ_min_ = −0.41 e Å^−^^3^
89 restraints	Absolute structure: Flack (1983), 4513 Friedel pairs
Primary atom site location: structure-invariant direct methods	Flack parameter: −0.01 (2)

### Fractional atomic coordinates and isotropic or equivalent isotropic displacement parameters (Å^2^)

**Table:**

	*x*	*y*	*z*	*U*_iso_*/*U*_eq_	
Sn1	0.49998 (2)	0.49997 (2)	1.00002 (2)	0.04882 (10)	
Sn2	−0.01462 (2)	0.60583 (2)	0.92111 (2)	0.04867 (10)	
S1	0.21418 (18)	0.20712 (15)	1.19010 (12)	0.0717 (4)	
S2	0.1212 (3)	0.4148 (2)	1.31804 (19)	0.1085 (7)	
S3	−0.39487 (16)	0.89805 (15)	0.73173 (13)	0.0716 (4)	
S4	−0.3672 (4)	0.7164 (3)	0.5797 (3)	0.1691 (17)	
O1	0.3468 (3)	0.3924 (4)	1.0643 (3)	0.0622 (9)	
O2	0.1838 (3)	0.5164 (4)	0.9783 (3)	0.0584 (9)	
O3	−0.2056 (3)	0.7115 (3)	0.8561 (3)	0.0627 (9)	
O4	−0.3317 (3)	0.5915 (4)	0.9437 (3)	0.0560 (9)	
N1	0.2493 (5)	0.1888 (5)	1.3937 (4)	0.0744 (13)	
N2	−0.3464 (10)	0.9487 (7)	0.5250 (5)	0.134 (4)	
C1	0.6270 (5)	0.3575 (5)	1.0974 (5)	0.0630 (14)	
H1A	0.6022	0.2803	1.1012	0.076*	
H1B	0.7144	0.3480	1.0619	0.076*	
C2	0.6259 (4)	0.3822 (4)	1.2114 (3)	0.0600 (13)	
C3	0.7312 (4)	0.4127 (5)	1.2428 (4)	0.089 (2)	
H3	0.8039	0.4166	1.1936	0.106*	
C4	0.7280 (6)	0.4374 (5)	1.3478 (5)	0.136 (4)	
H4	0.7984	0.4578	1.3688	0.163*	
C5	0.6194 (7)	0.4316 (5)	1.4213 (3)	0.123 (3)	
H5	0.6172	0.4481	1.4916	0.147*	
C6	0.5141 (5)	0.4011 (5)	1.3899 (3)	0.109 (3)	
H6	0.4414	0.3972	1.4391	0.131*	
C7	0.5173 (3)	0.3764 (5)	1.2850 (4)	0.0745 (17)	
H7	0.4469	0.3560	1.2639	0.089*	
C8	0.3774 (5)	0.6484 (5)	1.0777 (5)	0.0651 (14)	
H8A	0.3061	0.6859	1.0338	0.078*	
H8B	0.3411	0.6123	1.1474	0.078*	
C9	0.4324 (4)	0.7496 (3)	1.0979 (4)	0.0598 (17)	
C10	0.5249 (4)	0.7246 (3)	1.1711 (4)	0.0809 (18)	
H10	0.5523	0.6450	1.2075	0.097*	
C11	0.5763 (4)	0.8186 (5)	1.1901 (4)	0.107 (3)	
H11	0.6381	0.8019	1.2391	0.129*	
C12	0.5353 (5)	0.9376 (4)	1.1359 (5)	0.106 (3)	
H12	0.5698	1.0005	1.1486	0.128*	
C13	0.4429 (5)	0.9626 (3)	1.0626 (4)	0.093 (2)	
H13	0.4155	1.0423	1.0263	0.112*	
C14	0.3915 (4)	0.8687 (4)	1.0436 (3)	0.0708 (18)	
H14	0.3296	0.8854	0.9946	0.085*	
C15	0.4934 (5)	0.4621 (5)	0.8392 (5)	0.0613 (13)	
H15A	0.4104	0.4456	0.8342	0.074*	
H15B	0.5030	0.5335	0.7859	0.074*	
C16	0.5989 (3)	0.3537 (4)	0.8154 (3)	0.0635 (15)	
C17	0.5921 (5)	0.2361 (4)	0.8673 (4)	0.078 (2)	
H17	0.5208	0.2248	0.9160	0.093*	
C18	0.6917 (7)	0.1354 (3)	0.8465 (5)	0.118 (3)	
H18	0.6872	0.0568	0.8813	0.142*	
C19	0.7982 (5)	0.1523 (5)	0.7738 (5)	0.129 (4)	
H19	0.8649	0.0849	0.7599	0.155*	
C20	0.8050 (4)	0.2698 (7)	0.7219 (4)	0.128 (4)	
H20	0.8762	0.2811	0.6732	0.153*	
C21	0.7053 (4)	0.3705 (5)	0.7427 (3)	0.0796 (18)	
H21	0.7099	0.4492	0.7079	0.096*	
C22	0.0666 (6)	0.7431 (5)	0.8139 (5)	0.0632 (14)	
H22A	0.0015	0.8201	0.8073	0.076*	
H22B	0.1378	0.7559	0.8465	0.076*	
C23	0.1147 (3)	0.7116 (4)	0.7022 (3)	0.0615 (13)	
C24	0.2466 (3)	0.6838 (4)	0.6700 (4)	0.0773 (17)	
H24	0.3047	0.6898	0.7160	0.093*	
C25	0.2918 (4)	0.6470 (5)	0.5690 (4)	0.108 (3)	
H25	0.3801	0.6284	0.5475	0.129*	
C26	0.2050 (6)	0.6381 (5)	0.5003 (3)	0.120 (3)	
H26	0.2353	0.6135	0.4327	0.144*	
C27	0.0731 (6)	0.6659 (6)	0.5325 (4)	0.110 (3)	
H27	0.0151	0.6599	0.4865	0.132*	
C28	0.0279 (3)	0.7027 (5)	0.6335 (4)	0.087 (2)	
H28	−0.0603	0.7213	0.6550	0.104*	
C29	−0.0347 (5)	0.4565 (5)	0.8470 (5)	0.0593 (13)	
H29A	−0.1040	0.4237	0.8901	0.071*	
H29B	−0.0643	0.4919	0.7760	0.071*	
C30	0.0768 (3)	0.3491 (3)	0.8305 (3)	0.0507 (14)	
C31	0.0646 (4)	0.2318 (4)	0.8786 (3)	0.0695 (18)	
H31	−0.0126	0.2207	0.9182	0.083*	
C32	0.1677 (5)	0.1309 (3)	0.8674 (4)	0.089 (2)	
H32	0.1596	0.0523	0.8996	0.107*	
C33	0.2831 (4)	0.1474 (4)	0.8082 (4)	0.095 (2)	
H33	0.3521	0.0799	0.8007	0.114*	
C34	0.2952 (3)	0.2648 (4)	0.7601 (3)	0.0807 (19)	
H34	0.3724	0.2758	0.7205	0.097*	
C35	0.1921 (4)	0.3656 (3)	0.7713 (3)	0.0643 (14)	
H35	0.2002	0.4442	0.7392	0.077*	
C36	−0.0906 (6)	0.6499 (5)	1.0801 (5)	0.0614 (13)	
H36A	−0.1839	0.6630	1.0875	0.074*	
H36B	−0.0568	0.5812	1.1347	0.074*	
C37	−0.0549 (4)	0.7638 (3)	1.0994 (3)	0.0590 (14)	
C38	−0.1172 (4)	0.8787 (4)	1.0485 (3)	0.079 (2)	
H38	−0.1827	0.8853	1.0052	0.095*	
C39	−0.0815 (5)	0.9838 (3)	1.0626 (4)	0.092 (2)	
H39	−0.1232	1.0607	1.0286	0.110*	
C40	0.0164 (5)	0.9740 (4)	1.1274 (4)	0.100 (3)	
H40	0.0403	1.0443	1.1368	0.120*	
C41	0.0787 (4)	0.8591 (5)	1.1783 (4)	0.097 (3)	
H41	0.1442	0.8525	1.2217	0.116*	
C42	0.0430 (3)	0.7540 (4)	1.1642 (3)	0.0714 (16)	
H42	0.0846	0.6771	1.1982	0.086*	
C43	0.2326 (5)	0.4219 (5)	1.0437 (4)	0.0537 (12)	
C44	0.1470 (6)	0.3360 (7)	1.0941 (5)	0.0790 (19)	
H44A	0.1204	0.3053	1.0359	0.095*	
H44B	0.0697	0.3836	1.1301	0.095*	
C45	0.1957 (6)	0.2699 (5)	1.3110 (4)	0.0643 (14)	
C46	0.3030 (8)	0.0580 (7)	1.3936 (6)	0.114 (3)	
H46A	0.3822	0.0314	1.4291	0.136*	
H46B	0.3222	0.0401	1.3196	0.136*	
C47	0.2029 (11)	−0.0070 (9)	1.4547 (8)	0.159 (4)	
H47A	0.1210	0.0250	1.4236	0.190*	
H47B	0.2302	−0.0947	1.4522	0.190*	
C48	0.1908 (11)	0.0177 (7)	1.5682 (6)	0.157 (5)	
H48A	0.1386	−0.0326	1.6158	0.188*	
H48B	0.2753	−0.0010	1.5945	0.188*	
C49	0.1281 (6)	0.1499 (7)	1.5662 (6)	0.106 (3)	
H49A	0.1060	0.1693	1.6397	0.128*	
H49B	0.0499	0.1715	1.5296	0.128*	
C50	0.2245 (7)	0.2179 (7)	1.5054 (5)	0.094 (2)	
H50A	0.1911	0.3058	1.5043	0.113*	
H50B	0.3040	0.1929	1.5404	0.113*	
C51	−0.3112 (4)	0.6835 (5)	0.8757 (4)	0.0492 (11)	
C52	−0.4235 (5)	0.7622 (6)	0.8183 (5)	0.0753 (17)	
H52A	−0.4947	0.7864	0.8726	0.090*	
H52B	−0.4506	0.7126	0.7747	0.090*	
C53	−0.3661 (9)	0.8566 (8)	0.6014 (5)	0.104 (3)	
C54	−0.3379 (7)	1.0693 (8)	0.5407 (7)	0.099 (3)	
H54A	−0.3553	1.0779	0.6172	0.119*	
H54B	−0.3995	1.1332	0.5002	0.119*	
C55	−0.2030 (8)	1.0779 (11)	0.4995 (7)	0.138 (4)	
H55A	−0.1412	1.0080	0.5332	0.166*	
H55B	−0.1864	1.1530	0.5147	0.166*	
C56	−0.1936 (11)	1.0785 (10)	0.3797 (7)	0.165 (5)	
H56A	−0.1136	1.0983	0.3444	0.198*	
H56B	−0.2656	1.1384	0.3480	0.198*	
C57	−0.1970 (9)	0.9511 (10)	0.3672 (8)	0.154 (4)	
H57A	−0.1784	0.9396	0.2913	0.185*	
H57B	−0.1344	0.8903	0.4093	0.185*	
C58	−0.3305 (10)	0.9407 (11)	0.4087 (7)	0.154 (4)	
H58A	−0.3928	1.0068	0.3709	0.184*	
H58B	−0.3438	0.8627	0.3975	0.184*	

### Atomic displacement parameters (Å^2^)

**Table:**

	*U*^11^	*U*^22^	*U*^33^	*U*^12^	*U*^13^	*U*^23^
Sn1	0.04022 (18)	0.0499 (2)	0.0563 (2)	−0.01081 (16)	−0.00841 (15)	−0.00493 (17)
Sn2	0.0437 (2)	0.0493 (2)	0.0570 (2)	−0.01849 (17)	−0.00312 (16)	−0.00898 (17)
S1	0.1022 (11)	0.0663 (9)	0.0563 (8)	−0.0390 (8)	−0.0048 (7)	−0.0097 (7)
S2	0.150 (2)	0.0718 (12)	0.1005 (15)	−0.0089 (12)	−0.0197 (13)	−0.0243 (10)
S3	0.0854 (10)	0.0612 (9)	0.0606 (9)	−0.0079 (7)	−0.0045 (7)	−0.0051 (7)
S4	0.245 (4)	0.163 (3)	0.154 (3)	−0.148 (3)	0.064 (3)	−0.089 (2)
O1	0.0495 (19)	0.067 (2)	0.071 (2)	−0.0237 (17)	−0.0126 (17)	0.0073 (18)
O2	0.0448 (17)	0.068 (2)	0.065 (2)	−0.0227 (16)	−0.0094 (15)	−0.0014 (19)
O3	0.0461 (19)	0.060 (2)	0.082 (3)	−0.0172 (17)	−0.0094 (17)	−0.0016 (18)
O4	0.0423 (17)	0.066 (2)	0.062 (2)	−0.0166 (15)	−0.0087 (15)	−0.0063 (18)
N1	0.080 (3)	0.083 (4)	0.057 (3)	−0.020 (3)	−0.007 (2)	−0.003 (3)
N2	0.227 (10)	0.157 (8)	0.060 (4)	−0.126 (8)	0.020 (5)	−0.032 (4)
C1	0.048 (3)	0.061 (3)	0.073 (4)	−0.006 (2)	−0.012 (2)	0.001 (3)
C2	0.054 (3)	0.053 (3)	0.073 (4)	−0.012 (2)	−0.017 (2)	−0.001 (2)
C3	0.088 (4)	0.080 (4)	0.107 (5)	−0.031 (4)	−0.047 (4)	0.009 (4)
C4	0.163 (10)	0.119 (8)	0.157 (10)	−0.075 (7)	−0.099 (8)	0.026 (7)
C5	0.183 (10)	0.089 (6)	0.105 (7)	−0.018 (6)	−0.073 (7)	−0.010 (5)
C6	0.113 (6)	0.115 (7)	0.080 (5)	−0.002 (5)	−0.009 (5)	0.001 (5)
C7	0.076 (4)	0.071 (4)	0.075 (4)	−0.018 (3)	−0.019 (3)	0.005 (3)
C8	0.043 (2)	0.067 (4)	0.083 (4)	−0.007 (2)	0.000 (2)	−0.019 (3)
C9	0.045 (3)	0.069 (4)	0.063 (4)	−0.007 (3)	0.005 (2)	−0.020 (3)
C10	0.090 (4)	0.065 (4)	0.086 (4)	0.002 (3)	−0.030 (4)	−0.021 (3)
C11	0.094 (5)	0.114 (6)	0.125 (7)	0.006 (5)	−0.039 (5)	−0.071 (6)
C12	0.107 (6)	0.082 (5)	0.145 (8)	−0.032 (5)	0.010 (5)	−0.060 (5)
C13	0.100 (5)	0.063 (4)	0.115 (6)	−0.015 (4)	−0.009 (4)	−0.017 (4)
C14	0.074 (4)	0.056 (4)	0.080 (5)	−0.003 (3)	−0.013 (3)	−0.019 (4)
C15	0.059 (3)	0.063 (3)	0.067 (3)	−0.019 (3)	−0.019 (3)	−0.008 (3)
C16	0.065 (3)	0.069 (4)	0.068 (4)	−0.019 (3)	−0.024 (3)	−0.020 (3)
C17	0.087 (5)	0.065 (5)	0.092 (6)	−0.028 (4)	−0.030 (4)	−0.010 (4)
C18	0.148 (8)	0.075 (5)	0.138 (8)	0.014 (5)	−0.072 (7)	−0.047 (5)
C19	0.129 (8)	0.115 (7)	0.149 (9)	0.037 (6)	−0.070 (7)	−0.084 (7)
C20	0.069 (4)	0.201 (12)	0.130 (7)	−0.010 (6)	−0.003 (4)	−0.105 (8)
C21	0.073 (4)	0.101 (5)	0.078 (4)	−0.025 (4)	−0.007 (3)	−0.040 (4)
C22	0.057 (3)	0.061 (3)	0.074 (4)	−0.023 (3)	−0.003 (3)	−0.005 (3)
C23	0.053 (3)	0.058 (3)	0.071 (4)	−0.018 (2)	−0.004 (2)	0.001 (3)
C24	0.058 (3)	0.073 (4)	0.092 (5)	−0.021 (3)	0.007 (3)	0.005 (3)
C25	0.089 (5)	0.095 (6)	0.117 (7)	−0.012 (4)	0.036 (5)	−0.009 (5)
C26	0.138 (8)	0.127 (7)	0.081 (6)	−0.025 (6)	0.028 (5)	−0.014 (5)
C27	0.143 (8)	0.133 (8)	0.062 (5)	−0.048 (6)	−0.016 (5)	−0.009 (5)
C28	0.071 (4)	0.103 (6)	0.080 (5)	−0.019 (4)	−0.013 (3)	0.006 (4)
C29	0.054 (3)	0.056 (3)	0.077 (4)	−0.018 (2)	−0.015 (2)	−0.019 (3)
C30	0.054 (3)	0.049 (3)	0.055 (3)	−0.014 (2)	−0.016 (2)	−0.012 (3)
C31	0.080 (4)	0.064 (5)	0.064 (4)	−0.017 (3)	−0.014 (3)	0.000 (3)
C32	0.114 (6)	0.068 (4)	0.081 (5)	−0.003 (4)	−0.030 (4)	−0.011 (3)
C33	0.091 (5)	0.087 (5)	0.095 (5)	0.028 (4)	−0.037 (4)	−0.033 (4)
C34	0.063 (4)	0.108 (6)	0.082 (4)	−0.021 (3)	−0.011 (3)	−0.042 (4)
C35	0.055 (3)	0.075 (4)	0.071 (4)	−0.021 (3)	−0.005 (2)	−0.027 (3)
C36	0.058 (3)	0.068 (4)	0.064 (3)	−0.026 (3)	0.001 (2)	−0.015 (3)
C37	0.061 (3)	0.066 (4)	0.055 (3)	−0.027 (3)	0.011 (3)	−0.018 (3)
C38	0.099 (6)	0.070 (5)	0.072 (5)	−0.023 (5)	−0.011 (4)	−0.015 (4)
C39	0.128 (6)	0.077 (5)	0.083 (5)	−0.053 (4)	−0.001 (4)	−0.012 (4)
C40	0.116 (6)	0.096 (6)	0.110 (6)	−0.066 (5)	0.030 (5)	−0.048 (5)
C41	0.065 (4)	0.143 (8)	0.106 (6)	−0.042 (4)	0.005 (4)	−0.066 (6)
C42	0.054 (3)	0.088 (4)	0.078 (4)	−0.015 (3)	−0.004 (3)	−0.031 (3)
C43	0.051 (2)	0.071 (3)	0.049 (3)	−0.029 (2)	−0.006 (2)	−0.012 (2)
C44	0.080 (4)	0.112 (5)	0.063 (4)	−0.060 (4)	−0.024 (3)	0.004 (3)
C45	0.077 (3)	0.069 (3)	0.054 (3)	−0.032 (3)	−0.006 (2)	−0.006 (3)
C46	0.111 (5)	0.111 (6)	0.102 (6)	0.011 (5)	−0.027 (4)	−0.012 (5)
C47	0.197 (9)	0.123 (7)	0.159 (8)	−0.056 (7)	−0.004 (7)	−0.014 (6)
C48	0.176 (8)	0.167 (9)	0.119 (7)	−0.054 (7)	−0.005 (6)	0.012 (6)
C49	0.085 (4)	0.153 (7)	0.079 (4)	−0.032 (5)	0.004 (4)	−0.016 (5)
C50	0.102 (5)	0.117 (5)	0.066 (4)	−0.031 (4)	−0.019 (3)	−0.009 (4)
C51	0.042 (2)	0.052 (3)	0.054 (3)	−0.007 (2)	−0.0028 (19)	−0.014 (2)
C52	0.052 (3)	0.087 (4)	0.075 (4)	−0.013 (3)	−0.005 (3)	0.014 (3)
C53	0.134 (6)	0.140 (7)	0.069 (4)	−0.093 (6)	0.023 (4)	−0.036 (5)
C54	0.102 (5)	0.098 (5)	0.083 (5)	−0.001 (4)	0.001 (4)	−0.013 (4)
C55	0.150 (7)	0.147 (7)	0.138 (7)	−0.061 (6)	0.006 (6)	−0.054 (6)
C56	0.180 (9)	0.156 (9)	0.144 (8)	−0.046 (7)	0.030 (7)	−0.009 (7)
C57	0.163 (8)	0.177 (8)	0.113 (6)	−0.029 (7)	0.034 (6)	−0.051 (6)
C58	0.169 (8)	0.196 (9)	0.121 (7)	−0.075 (7)	−0.009 (6)	−0.048 (6)

### Geometric parameters (Å, °)

**Table:**

Sn1—C1	2.146 (5)	C23—C28	1.3900
Sn1—C8	2.153 (5)	C24—C25	1.3900
Sn1—C15	2.152 (5)	C24—H24	0.9300
Sn1—O1	2.268 (3)	C25—C26	1.3900
Sn1—O4^i^	2.276 (3)	C25—H25	0.9300
Sn2—C22	2.144 (5)	C26—C27	1.3900
Sn2—C29	2.146 (5)	C26—H26	0.9300
Sn2—C36	2.143 (5)	C27—C28	1.3900
Sn2—O2	2.272 (3)	C27—H27	0.9300
Sn2—O3	2.277 (4)	C28—H28	0.9300
S1—C45	1.757 (6)	C29—C30	1.499 (6)
S1—C44	1.784 (7)	C29—H29A	0.9700
S2—C45	1.651 (6)	C29—H29B	0.9700
S3—C53	1.748 (7)	C30—C31	1.3900
S3—C52	1.787 (6)	C30—C35	1.3900
S4—C53	1.668 (8)	C31—C32	1.3900
O1—C43	1.239 (6)	C31—H31	0.9300
O2—C43	1.270 (6)	C32—C33	1.3900
O3—C51	1.236 (6)	C32—H32	0.9300
O4—C51	1.268 (6)	C33—C34	1.3900
O4—Sn1^ii^	2.276 (3)	C33—H33	0.9300
N1—C45	1.344 (6)	C34—C35	1.3900
N1—C46	1.451 (7)	C34—H34	0.9300
N1—C50	1.471 (7)	C35—H35	0.9300
N2—C53	1.322 (7)	C36—C37	1.507 (6)
N2—C54	1.445 (8)	C36—H36A	0.9700
N2—C58	1.462 (8)	C36—H36B	0.9700
C1—C2	1.505 (7)	C37—C38	1.3900
C1—H1A	0.9700	C37—C42	1.3900
C1—H1B	0.9700	C38—C39	1.3900
C2—C3	1.3900	C38—H38	0.9300
C2—C7	1.3900	C39—C40	1.3900
C3—C4	1.3900	C39—H39	0.9300
C3—H3	0.9300	C40—C41	1.3900
C4—C5	1.3900	C40—H40	0.9300
C4—H4	0.9300	C41—C42	1.3900
C5—C6	1.3900	C41—H41	0.9300
C5—H5	0.9300	C42—H42	0.9300
C6—C7	1.3900	C43—C44	1.505 (7)
C6—H6	0.9300	C44—H44A	0.9700
C7—H7	0.9300	C44—H44B	0.9700
C8—C9	1.493 (6)	C46—C47	1.501 (5)
C8—H8A	0.9700	C46—H46A	0.9700
C8—H8B	0.9700	C46—H46B	0.9700
C9—C10	1.3900	C47—C48	1.484 (5)
C9—C14	1.3900	C47—H47A	0.9700
C10—C11	1.3900	C47—H47B	0.9700
C10—H10	0.9300	C48—C49	1.479 (5)
C11—C12	1.3900	C48—H48A	0.9700
C11—H11	0.9300	C48—H48B	0.9700
C12—C13	1.3900	C49—C50	1.490 (5)
C12—H12	0.9300	C49—H49A	0.9700
C13—C14	1.3900	C49—H49B	0.9700
C13—H13	0.9300	C50—H50A	0.9700
C14—H14	0.9300	C50—H50B	0.9700
C15—C16	1.491 (6)	C51—C52	1.496 (7)
C15—H15A	0.9700	C52—H52A	0.9700
C15—H15B	0.9700	C52—H52B	0.9700
C16—C17	1.3900	C54—C55	1.491 (5)
C16—C21	1.3900	C54—H54A	0.9700
C17—C18	1.3900	C54—H54B	0.9700
C17—H17	0.9300	C55—C56	1.491 (5)
C18—C19	1.3900	C55—H55A	0.9700
C18—H18	0.9300	C55—H55B	0.9700
C19—C20	1.3900	C56—C57	1.493 (5)
C19—H19	0.9300	C56—H56A	0.9700
C20—C21	1.3900	C56—H56B	0.9700
C20—H20	0.9300	C57—C58	1.483 (5)
C21—H21	0.9300	C57—H57A	0.9700
C22—C23	1.498 (7)	C57—H57B	0.9700
C22—H22A	0.9700	C58—H58A	0.9700
C22—H22B	0.9700	C58—H58B	0.9700
C23—C24	1.3900		
			
C1—Sn1—C15	114.1 (2)	Sn2—C29—H29A	107.0
C1—Sn1—C8	117.9 (2)	C30—C29—H29B	107.0
C15—Sn1—C8	127.4 (2)	Sn2—C29—H29B	107.0
C1—Sn1—O1	86.11 (19)	H29A—C29—H29B	106.7
C15—Sn1—O1	89.25 (19)	C31—C30—C35	120.0
C8—Sn1—O1	87.04 (18)	C31—C30—C29	118.8 (3)
C1—Sn1—O4^i^	88.16 (19)	C35—C30—C29	121.2 (3)
C15—Sn1—O4^i^	92.35 (18)	C30—C31—C32	120.0
C8—Sn1—O4^i^	96.38 (18)	C30—C31—H31	120.0
O1—Sn1—O4^i^	174.2 (1)	C32—C31—H31	120.0
C36—Sn2—C22	115.6 (2)	C31—C32—C33	120.0
C36—Sn2—C29	128.4 (2)	C31—C32—H32	120.0
C22—Sn2—C29	115.3 (2)	C33—C32—H32	120.0
C36—Sn2—O2	91.43 (19)	C34—C33—C32	120.0
C22—Sn2—O2	89.20 (19)	C34—C33—H33	120.0
C29—Sn2—O2	97.31 (17)	C32—C33—H33	120.0
C36—Sn2—O3	89.8 (2)	C33—C34—C35	120.0
C22—Sn2—O3	85.38 (19)	C33—C34—H34	120.0
C29—Sn2—O3	86.15 (17)	C35—C34—H34	120.0
O2—Sn2—O3	174.4 (1)	C34—C35—C30	120.0
C45—S1—C44	103.2 (3)	C34—C35—H35	120.0
C53—S3—C52	103.8 (4)	C30—C35—H35	120.0
C43—O1—Sn1	126.5 (3)	C37—C36—Sn2	111.3 (3)
C43—O2—Sn2	136.4 (3)	C37—C36—H36A	109.4
C51—O3—Sn2	127.9 (3)	Sn2—C36—H36A	109.4
C51—O4—Sn1^ii^	136.4 (3)	C37—C36—H36B	109.4
C45—N1—C46	126.1 (6)	Sn2—C36—H36B	109.4
C45—N1—C50	120.7 (6)	H36A—C36—H36B	108.0
C46—N1—C50	110.9 (5)	C38—C37—C42	120.0
C53—N2—C54	127.1 (6)	C38—C37—C36	119.6 (4)
C53—N2—C58	122.9 (7)	C42—C37—C36	120.3 (4)
C54—N2—C58	110.0 (7)	C37—C38—C39	120.0
C2—C1—Sn1	113.6 (3)	C37—C38—H38	120.0
C2—C1—H1A	108.8	C39—C38—H38	120.0
Sn1—C1—H1A	108.8	C38—C39—C40	120.0
C2—C1—H1B	108.8	C38—C39—H39	120.0
Sn1—C1—H1B	108.8	C40—C39—H39	120.0
H1A—C1—H1B	107.7	C39—C40—C41	120.0
C3—C2—C7	120.0	C39—C40—H40	120.0
C3—C2—C1	120.6 (4)	C41—C40—H40	120.0
C7—C2—C1	119.4 (4)	C42—C41—C40	120.0
C2—C3—C4	120.0	C42—C41—H41	120.0
C2—C3—H3	120.0	C40—C41—H41	120.0
C4—C3—H3	120.0	C41—C42—C37	120.0
C3—C4—C5	120.0	C41—C42—H42	120.0
C3—C4—H4	120.0	C37—C42—H42	120.0
C5—C4—H4	120.0	O1—C43—O2	124.8 (5)
C6—C5—C4	120.0	O1—C43—C44	117.9 (5)
C6—C5—H5	120.0	O2—C43—C44	117.2 (5)
C4—C5—H5	120.0	C43—C44—S1	116.9 (4)
C5—C6—C7	120.0	C43—C44—H44A	108.1
C5—C6—H6	120.0	S1—C44—H44A	108.1
C7—C6—H6	120.0	C43—C44—H44B	108.1
C6—C7—C2	120.0	S1—C44—H44B	108.1
C6—C7—H7	120.0	H44A—C44—H44B	107.3
C2—C7—H7	120.0	N1—C45—S2	125.0 (5)
C9—C8—Sn1	119.7 (3)	N1—C45—S1	113.2 (5)
C9—C8—H8A	107.4	S2—C45—S1	121.9 (3)
Sn1—C8—H8A	107.4	N1—C46—C47	106.8 (7)
C9—C8—H8B	107.4	N1—C46—H46A	110.4
Sn1—C8—H8B	107.4	C47—C46—H46A	110.4
H8A—C8—H8B	106.9	N1—C46—H46B	110.4
C10—C9—C14	120.0	C47—C46—H46B	110.4
C10—C9—C8	119.8 (4)	H46A—C46—H46B	108.6
C14—C9—C8	120.2 (4)	C48—C47—C46	105.3 (4)
C11—C10—C9	120.0	C48—C47—H47A	110.7
C11—C10—H10	120.0	C46—C47—H47A	110.7
C9—C10—H10	120.0	C48—C47—H47B	110.7
C10—C11—C12	120.0	C46—C47—H47B	110.7
C10—C11—H11	120.0	H47A—C47—H47B	108.8
C12—C11—H11	120.0	C49—C48—C47	106.6 (5)
C13—C12—C11	120.0	C49—C48—H48A	110.4
C13—C12—H12	120.0	C47—C48—H48A	110.4
C11—C12—H12	120.0	C49—C48—H48B	110.4
C14—C13—C12	120.0	C47—C48—H48B	110.4
C14—C13—H13	120.0	H48A—C48—H48B	108.6
C12—C13—H13	120.0	C48—C49—C50	105.8 (4)
C13—C14—C9	120.0	C48—C49—H49A	110.6
C13—C14—H14	120.0	C50—C49—H49A	110.6
C9—C14—H14	120.0	C48—C49—H49B	110.6
C16—C15—Sn1	110.2 (3)	C50—C49—H49B	110.6
C16—C15—H15A	109.6	H49A—C49—H49B	108.7
Sn1—C15—H15A	109.6	N1—C50—C49	107.8 (5)
C16—C15—H15B	109.6	N1—C50—H50A	110.1
Sn1—C15—H15B	109.6	C49—C50—H50A	110.1
H15A—C15—H15B	108.1	N1—C50—H50B	110.1
C17—C16—C21	120.0	C49—C50—H50B	110.1
C17—C16—C15	120.1 (4)	H50A—C50—H50B	108.5
C21—C16—C15	119.9 (4)	O3—C51—O4	123.8 (4)
C18—C17—C16	120.0	O3—C51—C52	119.3 (5)
C18—C17—H17	120.0	O4—C51—C52	117.0 (4)
C16—C17—H17	120.0	C51—C52—S3	115.8 (4)
C17—C18—C19	120.0	C51—C52—H52A	108.3
C17—C18—H18	120.0	S3—C52—H52A	108.3
C19—C18—H18	120.0	C51—C52—H52B	108.3
C20—C19—C18	120.0	S3—C52—H52B	108.3
C20—C19—H19	120.0	H52A—C52—H52B	107.4
C18—C19—H19	120.0	N2—C53—S4	125.4 (6)
C19—C20—C21	120.0	N2—C53—S3	112.3 (6)
C19—C20—H20	120.0	S4—C53—S3	122.3 (4)
C21—C20—H20	120.0	N2—C54—C55	105.6 (7)
C20—C21—C16	120.0	N2—C54—H54A	110.6
C20—C21—H21	120.0	C55—C54—H54A	110.6
C16—C21—H21	120.0	N2—C54—H54B	110.6
C23—C22—Sn2	114.3 (3)	C55—C54—H54B	110.6
C23—C22—H22A	108.7	H54A—C54—H54B	108.8
Sn2—C22—H22A	108.7	C54—C55—C56	105.3 (4)
C23—C22—H22B	108.7	C54—C55—H55A	110.7
Sn2—C22—H22B	108.7	C56—C55—H55A	110.7
H22A—C22—H22B	107.6	C54—C55—H55B	110.7
C24—C23—C28	120.0	C56—C55—H55B	110.7
C24—C23—C22	120.0 (3)	H55A—C55—H55B	108.8
C28—C23—C22	119.9 (3)	C55—C56—C57	105.3 (4)
C25—C24—C23	120.0	C55—C56—H56A	110.7
C25—C24—H24	120.0	C57—C56—H56A	110.7
C23—C24—H24	120.0	C55—C56—H56B	110.7
C24—C25—C26	120.0	C57—C56—H56B	110.7
C24—C25—H25	120.0	H56A—C56—H56B	108.8
C26—C25—H25	120.0	C58—C57—C56	104.8 (4)
C27—C26—C25	120.0	C58—C57—H57A	110.8
C27—C26—H26	120.0	C56—C57—H57A	110.8
C25—C26—H26	120.0	C58—C57—H57B	110.8
C26—C27—C28	120.0	C56—C57—H57B	110.8
C26—C27—H27	120.0	H57A—C57—H57B	108.9
C28—C27—H27	120.0	N2—C58—C57	107.0 (7)
C27—C28—C23	120.0	N2—C58—H58A	110.3
C27—C28—H28	120.0	C57—C58—H58A	110.3
C23—C28—H28	120.0	N2—C58—H58B	110.3
C30—C29—Sn2	121.3 (3)	C57—C58—H58B	110.3
C30—C29—H29A	107.0	H58A—C58—H58B	108.6
			
C1—Sn1—O1—C43	178.3 (5)	C29—Sn2—C36—C37	−179.3 (3)
C15—Sn1—O1—C43	−67.5 (5)	O2—Sn2—C36—C37	80.2 (4)
C8—Sn1—O1—C43	60.0 (5)	O3—Sn2—C36—C37	−94.4 (4)
C36—Sn2—O2—C43	66.6 (5)	Sn2—C36—C37—C38	74.1 (4)
C22—Sn2—O2—C43	−177.8 (5)	Sn2—C36—C37—C42	−103.4 (4)
C29—Sn2—O2—C43	−62.5 (5)	C36—C37—C38—C39	−177.6 (4)
C36—Sn2—O3—C51	−69.4 (5)	C36—C37—C42—C41	177.5 (4)
C22—Sn2—O3—C51	174.9 (5)	Sn1—O1—C43—O2	3.2 (8)
C29—Sn2—O3—C51	59.2 (5)	Sn1—O1—C43—C44	179.3 (4)
C15—Sn1—C1—C2	−177.2 (3)	Sn2—O2—C43—O1	−178.1 (4)
C8—Sn1—C1—C2	−5.2 (5)	Sn2—O2—C43—C44	5.8 (8)
O1—Sn1—C1—C2	−89.8 (4)	O1—C43—C44—S1	6.2 (8)
O4^i^—Sn1—C1—C2	91.0 (4)	O2—C43—C44—S1	−177.4 (4)
Sn1—C1—C2—C3	−108.5 (4)	C45—S1—C44—C43	81.4 (5)
Sn1—C1—C2—C7	70.4 (4)	C46—N1—C45—S2	172.9 (6)
C1—C2—C3—C4	178.9 (4)	C50—N1—C45—S2	11.4 (8)
C1—C2—C7—C6	−178.9 (4)	C46—N1—C45—S1	−8.0 (8)
C1—Sn1—C8—C9	78.6 (5)	C50—N1—C45—S1	−169.5 (4)
C15—Sn1—C8—C9	−110.6 (4)	C44—S1—C45—N1	−175.5 (4)
O1—Sn1—C8—C9	162.6 (4)	C44—S1—C45—S2	3.7 (5)
O4^i^—Sn1—C8—C9	−12.7 (4)	C45—N1—C46—C47	−100.7 (7)
Sn1—C8—C9—C10	−66.2 (5)	C50—N1—C46—C47	62.3 (8)
Sn1—C8—C9—C14	113.8 (4)	N1—C46—C47—C48	−65.1 (8)
C8—C9—C10—C11	180.0 (4)	C46—C47—C48—C49	69.7 (8)
C8—C9—C14—C13	−180.0 (4)	C47—C48—C49—C50	−68.5 (7)
C1—Sn1—C15—C16	−11.3 (4)	C45—N1—C50—C49	102.6 (7)
C8—Sn1—C15—C16	177.6 (3)	C46—N1—C50—C49	−61.5 (7)
O1—Sn1—C15—C16	−96.7 (4)	C48—C49—C50—N1	62.7 (7)
O4^i^—Sn1—C15—C16	77.7 (4)	Sn2—O3—C51—O4	4.9 (7)
Sn1—C15—C16—C17	71.6 (4)	Sn2—O3—C51—C52	−175.9 (4)
Sn1—C15—C16—C21	−107.2 (3)	Sn1^ii^—O4—C51—O3	−176.3 (4)
C15—C16—C17—C18	−178.7 (4)	Sn1^ii^—O4—C51—C52	4.5 (8)
C15—C16—C21—C20	178.7 (4)	O3—C51—C52—S3	−4.3 (8)
C36—Sn2—C22—C23	−178.0 (4)	O4—C51—C52—S3	175.0 (4)
C29—Sn2—C22—C23	−6.9 (5)	C53—S3—C52—C51	99.4 (6)
O2—Sn2—C22—C23	90.8 (4)	C54—N2—C53—S4	−176.5 (8)
O3—Sn2—C22—C23	−90.4 (4)	C58—N2—C53—S4	4.0 (16)
Sn2—C22—C23—C24	−110.2 (4)	C54—N2—C53—S3	4.6 (14)
Sn2—C22—C23—C28	65.8 (5)	C58—N2—C53—S3	−174.9 (8)
C22—C23—C24—C25	175.9 (4)	C52—S3—C53—N2	178.2 (7)
C36—Sn2—C29—C30	−107.8 (4)	C52—S3—C53—S4	−0.8 (7)
C22—Sn2—C29—C30	82.4 (5)	C53—N2—C54—C55	115.4 (11)
O2—Sn2—C29—C30	−10.1 (4)	C58—N2—C54—C55	−65.0 (10)
O3—Sn2—C29—C30	165.5 (4)	N2—C54—C55—C56	66.8 (8)
Sn2—C29—C30—C31	121.0 (3)	C54—C55—C56—C57	−70.3 (9)
Sn2—C29—C30—C35	−57.1 (5)	C55—C56—C57—C58	69.0 (8)
C29—C30—C31—C32	−178.1 (4)	C53—N2—C58—C57	−115.4 (10)
C29—C30—C35—C34	178.1 (4)	C54—N2—C58—C57	65.0 (11)
C22—Sn2—C36—C37	−9.6 (5)	C56—C57—C58—N2	−65.2 (10)

Symmetry codes: (i) *x*+1, *y*, *z*; (ii) *x*−1, *y*, *z*.

## References

[bb1] Barbour, L. J. (2001). *J. Supramol. Chem.* **1**, 189–191.

[bb2] Bruker (2009). *APEX2* and *SAINT* Bruker AXS Inc., Madison, Wisconsin, USA.

[bb3] Flack, H. D. (1983). *Acta Cryst.* A**39**, 876–881.

[bb4] Nachmias, G. (1952). *Ann. Chim.* **12**, 584–631.

[bb5] Ng, S. W., Chen, W. & Kumar Das, V. G. (1988). *J. Organomet. Chem.* **345**, 59–64.

[bb6] Ng, S. W. & Kumar Das, V. G. (1991). *J. Organomet. Chem.* **409**, 143–156.

[bb7] Sheldrick, G. M. (1996). *SADABS* University of Göttingen, Germany.

[bb8] Sheldrick, G. M. (2008). *Acta Cryst.* A**64**, 112–122. 10.1107/S0108767307043930 18156677

[bb9] Sisido, K., Takeda, Y. & Kinugawa, Z. (1961). *J. Am. Chem. Soc.* **83**, 538–541.

[bb10] Westrip, S. P. (2010). *J. Appl. Cryst.* **43**, 920–925.

